# Association of birthweight and penetrance of diabetes in individuals with *HNF4A*-MODY: a cohort study

**DOI:** 10.1007/s00125-021-05581-6

**Published:** 2021-10-07

**Authors:** Jonathan M. Locke, Petra Dusatkova, Kevin Colclough, Alice E. Hughes, John M. Dennis, Beverley Shields, Sarah E. Flanagan, Maggie H. Shepherd, Emma L. Dempster, Andrew T. Hattersley, Michael N. Weedon, Stepanka Pruhova, Kashyap A. Patel

**Affiliations:** 1 Institute of Biomedical & Clinical Science, College of Medicine & Health, University of Exeter, Exeter, UK; 2 Department of Pediatrics, Second Faculty of Medicine, Charles University and University Hospital Motol, Prague, Czech Republic; 3 Exeter Genomics Laboratory, Royal Devon and Exeter NHS Foundation Trust, Exeter, UK; 4 Exeter NIHR Clinical Research Facility, Royal Devon and Exeter NHS Foundation Trust, Exeter, UK

**Keywords:** Birthweight, Hepatocyte nuclear factor-4 alpha (HNF4A), Maturity-onset diabetes of the young (MODY), Penetrance

*To the Editor*: Mutations in *HNF4A*, which encodes hepatocyte nuclear factor-4 α, cause maturity-onset diabetes of the young (MODY) [1]. *HNF4A*-MODY is also associated with congenital hyperinsulinism (CHI) and neonatal hypoglycaemia [2]. Our understanding of the transition from hyperinsulinism to diabetes is limited. One hypothesis is higher fetal insulin secretion triggers apoptosis and results in accelerated postnatal beta cell failure. Alternatively, HNF4A deficiency could cause distinct transcriptional defects in early and late life, leading to the contrasting insulin phenotypes [2, 3]. These two hypotheses differ as to whether hypersecretion of insulin in utero precipitates beta cell failure later in life.

Fetal insulin secretion is known to be a major determinant of birthweight, particularly in humans [4, 5]. Our analysis of individuals with complete absence of fetal insulin secretion, due to mutations in the insulin gene or pancreatic agenesis, have shown they are half the normal birthweight [5]. Additionally, in individuals with *HNF4A* mutations, macrosomia has been reported as always occurring in those with neonatal hyperinsulinaemic hypoglycaemia [2]. Thus, birthweight may be a useful bioassay of fetal insulin secretion. In this study we sought to determine the association between birthweight and penetrance of diabetes in individuals with *HNF4A* mutations, which could provide insights into the relationship between fetal and adult insulin secretory capacity.

Data from genetic testing referral forms was extracted for all individuals with birthweight data and a rare (absent from gnomAD v2.1.1 [5]) pathogenic *HNF4A* mutation (Molecular Genetics laboratory, Exeter, UK [*n* = 204], and Motol University Hospital, Prague, Czech Republic [*n* = 59]). Due to a lack of population-level sequencing data, which is needed to confidently define variant penetrance and pathogenicity, we then excluded a small number of individuals self-reported as non-White (*n* = 7). Individuals aged <7 years at study (*n* = 63) were also removed due to lack of diabetes presenting. Finally, for accurate determination of corrected birthweight, a small number of individuals self-reported as a twin (*n* = 4) or born ≤32 weeks gestation (*n* = 3) were removed. This resulted in the cohort presented here (*n* = 186, see electronic supplementary material [ESM] Table 1 for clinical characteristics). The characteristics of our cohort meant we calculated we had >80% power to detect a HR ≥ 1.43 or ≤ 0.70. BMI for children was adjusted to adult equivalent using the Child Growth Foundation reference standards [6]. Birthweights were corrected for gestation and sex using British 1990/UK-WHO growth standards [7] and calculated using the *zanthro* package [8]. Statistical analyses were performed using Stata v16 (StataCorp, Texas, USA). This study had ethical approval from the North Wales Research Ethics Committee (17/WA/0327) and Motol University Hospital Ethics Commitee.

We first sought to assess the impact of maternal diabetes and an *HNF4A* mutation on birthweight. The birthweight of individuals with a mutation was higher than that of family members without the mutation (4.17 kg [IQR = 3.60–4.71], *n* = 186 vs 3.66 kg [IQR = 3.30–4.18], *n* = 31; *p*< 0.001). The results were similar when analysis was restricted to individuals born to mothers not reported to have diabetes (4.03 kg [IQR = 3.64–4.56], *n* = 72 vs 3.44 kg [IQR = 3.24–3.85], *n* = 20; *p* = 0.001) (Fig. 1a). Furthermore, for individuals with a mutation, those born to a mother with diabetes during pregnancy had an even higher birthweight than those born to a mother never reported to have diabetes (4.47 kg [IQR = 3.76–4.88], *n* = 55 vs 4.03 kg [IQR = 3.64–4.56], *n* = 72; *p* = 0.04) (Fig. 1a).

**Fig. 1:**
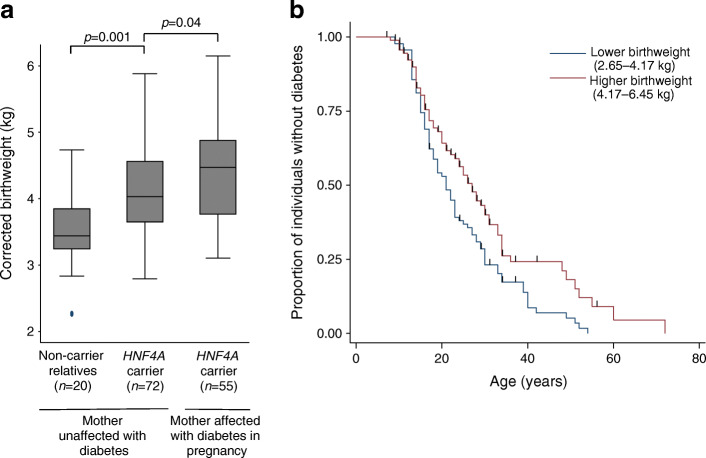
*HNF4A* mutations are associated with a maternal glucose-independent increase in birthweight and a higher birthweight is associated with relative protection from diabetes. (**a**) Sex and gestation-corrected birthweights for individuals with *HNF4A* mutations, split by presence of maternal diabetes during pregnancy, and non-carrier relatives. *p* values were calculated by Mann–Whitney *U* tests. The horizontal line within each box represents the median, and the top and bottom of the box represent the 75th and 25th percentiles, respectively. The whiskers indicate the maximum and minimum values, excluding outliers. Filled circles indicate outliers. (**b**) Kaplan–Meier plot of survival from diabetes split by median birthweight for individuals with *HNF4A* mutations (*n* = 186)

To assess the impact of birthweight on penetrance, we performed univariable Cox proportional-hazards survival analysis and found each per-kg increase was associated with a 22% reduction in diabetes diagnoses (HR = 0.78 [95% CI = 0.62, 0.97]; *p* = 0.03) (Table 1). For those individuals with a birthweight less than 4.17 kg (cohort median), 50% had developed diabetes by the age of 22 years, whereas for individuals with a birthweight more than 4.17 kg, 50% had developed diabetes by the age of 30 years (Fig. 1b). We observed similar results when analysis was limited to individuals with protein-truncating variants (HR = 0.64, 95% CI = 0.42, 0.98, *p* = 0.04), or a likely paternally inherited mutation (father reported to have diabetes and/or mutation and mother reported not to have diabetes) (HR = 0.63, 95% CI = 0.40, 0.99, *p* = 0.05).

**Table 1:** Hazard ratios for factors associated with penetrance of *HNF4A*-MODY identified by Cox proportional-hazards survival regression analyses

	Univariable		Multivariable	
	HR (95% CI)	*p*	HR (95% CI)	*p*
Female	1.40 (1.00, 1.98)	0.053	1.60 (1.09, 2.34)	0.015
Proband	2.47 (1.75, 3.48)	<0.001	2.98 (2.02, 4.40)	<0.001
Mother with diabetes during pregnancy	2.13 (1.42, 3.19)	<0.001	3.07 (2.00, 4.71)	<0.001
Corrected birthweight (per kg increase)	0.78 (0.62, 0.97)	0.03	0.70 (0.54, 0.91)	0.008

To assess if birthweight is independently associated with penetrance, we conducted multivariable Cox proportional-hazards survival analyses adjusting for other factors affecting penetrance. To identify factors associated with penetrance of *HNF4A*-MODY, we compared the characteristics of individuals with or without diabetes at 20 years of age (median age of diabetes diagnosis for cohort). Besides lower birthweight, being a proband, female, and born to a mother with diabetes during pregnancy was associated with earlier onset (all *p*< 0.05) (ESM Table 2). After adjusting for these variables, the effect of birthweight on penetrance remained independent, with each per-kg increase associated with a 30% reduction in diabetes diagnoses (HR = 0.70 [95% CI = 0.54, 0.91]; *p* = 0.008, Table 1).

We have found that, amongst individuals with highly penetrant *HNF4A* mutations, a higher birthweight is associated with reduced penetrance of diabetes. We speculate that this reflects a correlation between an individual’s fetal and adult insulin secretory capacity, such that there is a lesser reduction in adult beta cell function for individuals with an *HNF4A* mutation and a higher birthweight. Direct measurement of perinatal glucose/insulin levels and longitudinal studies of beta cell function would enable firmer mechanistic conclusions, but these studies are inherently difficult and costly to perform.

We have recently reported that transfer to sulfonylureas is not universally successful for individuals with *HNF4A*-MODY, and that BMI and diabetes duration are predictors of transfer success [9]. Given the results presented here, we believe studies investigating treatment response with respect to birthweight and mother’s diabetes status during pregnancy are warranted.

We previously reported a hypomorphic *HNF4A* missense mutation (p.R114W) associated with no increase in birthweight and reduced penetrance [10]. The requirement for mutations in this cohort to be absent from gnomAD should have limited the inclusion of individuals with similar, relatively common, hypomorphic mutations. If some hypomorphic mutations have been included, and act similarly to p.R114W, then their presence would result in an underestimation of the modifying effect of birthweight on penetrance. Future analyses of penetrance in population cohorts consisting of millions of sequenced individuals will be important for refining the effect size estimate.

In conclusion, this is one of the first and largest studies investigating the factors affecting penetrance of *HNF4A*-MODY, identifying birthweight as a modifier of potential prognostic and therapeutic relevance.

## Supplementary Information

ESM (PDF 157 kb)

## Data Availability

The datasets generated and/or analysed during the current study are available from the corresponding authors upon reasonable request.
