# Supplementary material for: Association of birthweight and penetrance of diabetes in individuals with HNF4A-MODY: a cohort study
Source: Diabetologia. Author manuscript; Available in PMC 2022 Jan 1. (PMC8660751; doi:10.1007/s00125-021-05581-6)
Supplement: Supplementary Material [file EMS138149-supplement-Supplementary_Material.docx]

| *n* | 186 |
| --- | --- |
| Birthweight adjusted for sex and gestational age, z score | 1.32 (0.11-2.45) |
| Corrected birthweight, kg | 4.17 (3.60-4.71) |
| Females (%) | 116 (62) |
| Age at referral, years | 30 (18-38) |
| Affected with diabetes (%) | 144 (77) |
| Age at diabetes diagnosis | 20 (15-29) |
| BMI at referral ^a^ | 23 (22-26) |
| Probands (%) | 91 (49) |
| Mother with diabetes (%) ^b^ | 104 (59) |
| Father with diabetes (%) ^b^ | 74 (42) |
| Protein truncating variants (PTV) (%) ^c^ | 55 (30) |
| Mutation affects all isoforms (%) | 137 (74) |

**ESM Table 1. Clinical characteristics of cohort.** Continuous variables are presented as median (IQR). ^a^ data available for n=125 ^b^ data available for n=176 ^c^ PTV includes essential splice site, nonsense, frameshift and multi-exon deletion variants

|  | **Diabetes by 20 years** | **Without diabetes at 20 years** | ***p*** |
| --- | --- | --- | --- |
| *n* | 72 | 96 |  |
| Corrected birthweight, kg | 3.96 (3.43-4.51) | 4.25 (3.75-4.72) | 0.047 |
| Females (%) | 53 (74) | 53 (55) | 0.016 |
| Age at referral (years) | 20 (14-33) | 35 (29-48) | <0.001 |
| BMI at referral ^a^ | 23 (21-27) | 24 (22-26) | 0.34 |
| Probands (%) | 49 (68) | 38 (40) | <0.001 |
| Mother with diabetes (%) ^b^ | 46 (65) | 48 (53) | 0.15 |
| Father with diabetes (%) ^b^ | 26 (37) | 44 (48) | 0.15 |
| Mother with diabetes during pregnancy (%) ^c^ | 30 (50) | 18 (22) | 0.001 |
| Protein truncating variants (PTV) (%) ^d^ | 25 (35) | 24 (25) | 0.18 |
| Mutation affects all isoforms (%) | 54 (75) | 71 (74) | 1.00 |
| Exeter cohort (%) | 56 (78) | 71 (74) | 0.59 |

**ESM Table 2. Clinical and genetic characteristics of cohort split by penetrance of diabetes**. Continuous variables are presented as median (IQR). Mann-Whitney U and Fisher’s Exact test used where appropriate. ^a^ data available for n=119 ^b^ data available for n=162 ^c^ data available for n=142 ^d^ PTV includes essential splice site, nonsense, frameshift and multi-exon deletion variants.
