# Supplementary material for: Association of birthweight and penetrance of diabetes in individuals with HNF4A-MODY: a cohort study
Source: Diabetologia. 2021 Oct 7;65(1):246–9. doi: 10.1007/s00125-021-05581-6 (PMC8660751; doi:10.1007/s00125-021-05581-6)
Supplement: Supplementary file 1 [file 125_2021_5581_MOESM1_ESM.pdf]

n	186
Birthweight adjusted for sex and gestational age, z score	1.32 (0.11-2.45)
Corrected birthweight, kg	4.17 (3.60-4.71)
Females (%)	116 (62)
Age at referral, years	30 (18-38)
Affected with diabetes (%)	144 (77)
Age at diabetes diagnosis	20 (15-29)
BMI at referral [a]	23 (22-26)
Probands (%)	91 (49)
Mother with diabetes (%) [b]	104 (59)
Father with diabetes (%) [b]	74 (42)
Protein truncating variants (PTV) (%) [c]	55 (30)
Mutation affects all isoforms (%)	137 (74)

ESM Table 1. Clinical characteristics of cohort. Continuous variables are presented as median (IQR). [a] data available for n=125 [b] data available for n=176 [c] PTV includes essential splice site, nonsense, frameshift and multi-exon deletion variants

	Diabetes by 20 years	Without diabetes at 20 years	*p*
n	72	96	
Corrected birthweight, kg	3.96 (3.43-4.51)	4.25 (3.75-4.72)	0.047
Females (%)	53 (74)	53 (55)	0.016
Age at referral (years)	20 (14-33)	35 (29-48)	<0.001
BMI at referral [a]	23 (21-27)	24 (22-26)	0.34
Probands (%)	49 (68)	38 (40)	<0.001
Mother with diabetes (%) [b]	46 (65)	48 (53)	0.15
Father with diabetes (%) [b]	26 (37)	44 (48)	0.15
Mother with diabetes during pregnancy (%) [c]	30 (50)	18 (22)	0.001
Protein truncating variants (PTV) (%) [d]	25 (35)	24 (25)	0.18
Mutation affects all isoforms (%)	54 (75)	71 (74)	1.00
Exeter cohort (%)	56 (78)	71 (74)	0.59

ESM Table 2. Clinical and genetic characteristics of cohort split by penetrance of diabetes. Continuous variables are presented as median (IQR). Mann-Whitney U and Fisher's Exact test used where appropriate. [a] data available for n=119 [b] data available for n=162 [c] data available for n=142 [d] PTV includes essential splice site, nonsense, frameshift and multi-exon deletion variants.
